# Expanding Universe Illusion

**DOI:** 10.1177/2041669519853848

**Published:** 2019-06-05

**Authors:** David Phillips, Priscilla Heard, Christopher W. Tyler

**Affiliations:** Hemel Hempstead, UK; University of the West of England, Bristol, UK; City University of London, UK

**Keywords:** visual illusion, motion, induced motion, optic flow

## Abstract

We present a new induced movement illusion from global expansion or contraction in a triangular region filled with rising or falling textures. Objective global expansion or contraction induces lateral movement in the oblique edges of the triangle. The effects may be due to common and relative movements operating within a single texture.

In this new illusion of induced movement, the oblique edges of a triangular patch of moving texture appear to expand (or contract) laterally as the texture within it expands (or contracts). In Movie 1, there is a rising (or falling) background to the triangle, which enhances effect for some observers, but is not essential.

**Movie 1.** The oblique edges of a triangular patch of moving texture appear to expand (or contract) laterally as the texture within it expands (or contracts). In movies 2 to 8, we show only rising textures, but opposite effects with falling ones should be assumed.

Nawrot and Sekuler (1990) studied the conditions in which test elements moved with or against their surround. The latter are more typical (e.g., Nakayama & Tyler, 1978), but in the illusion reported here, the oblique edges move with the direction of the inducing field. The illusion is an outgrowth of an effect reported by two of us in a study of certain novelty rings (Heard & Phillips, 2015) and enhanced in a shortlisted entry to the Best Visual Illusion of the Year Contest in 2017. With Tyler as a third collaborator, a different variant won Second Prize in the 2018 contest.

Reducing the illusion to its simplest elements, movement along the trajectories of two dotted lines induces illusory expansion or contraction of the distance between them depending on the direction of movement. Movie 2 shows that the more vertical stream can be vertical or oblique, and that effect is stronger if the array is made symmetrical.

**Movie 2.** The more vertical stream can be vertical or oblique, and effect is stronger if the array is made symmetrical.

The illusion is robust over a range of speeds but fails at high speed (Movie 3(a)). It becomes stronger as the angle between the streams increases (Movie 3(b)) but weaker with increasing separation (Movie 3(c)).

**Movie 3.** Dot velocity doubles at each stage in Movie 3(a), and illusion fails at high speed. It becomes stronger as the angle between the streams increases (Movie 3(b)) but weaker with increasing separation (Movie 3(c)).

Movie 4 shows the illusion with a texture of multiple dot streams, each dot still following a straight track, ranging from vertical at the centre of the triangle, to oblique along its edges. This variant of the illusion enables us to consider separately first the expanding and thinning of the texture as it rises within the triangle and secondly the illusory lateral movement of the oblique edges.

**Movie 4.** With a texture of multiple dot streams, each dot still follows a straight track, ranging from vertical at the centre of the triangle, to oblique along its edges. The dense texture reveals a more complex story.

The effect of expansion within the triangle can be isolated from the edge movement effect if the texture is made very sparse (Movie 5), so that the triangle edges are no longer apparent.

**Movie 5.** With a very sparse texture, triangle edges are no longer apparent and the character of the texture expansion is prominent.

The lateral expansion of the texture, especially near the source where it is almost explosive, is more salient than its vertical movement. In typical illusions of induced or relative movement (Anstis, 2018), such an effect might be attributable to the movement of a foreground object seen in the context of a rising background, as in Movie 5(a). Movement components shared between background and foreground are inhibited, whilst components that differ are exaggerated. But in Movie 5(b), there is no background. The effect appears driven by different movement components within the texture.

The illusory movement of the triangle edges seems similarly to depend on interaction between different elements within the texture. In Movie 6, the left half of the triangle is overturned, but with its movement reversed, to replace the triangle with a parallelogram with a central seam. The effect is now of a three-dimensional twist about the vertical seam line.

**Movie 6.** In this adapted variant, the parallelogram appears to twist in 3-D around a vertical seamline.

The illusory edge movement can be cancelled in various ways. If the edges of the triangle are occluded by a mask of circular apertures (Movie 7(a)), induced edge lateral movement vanishes, whilst within the circles, the texture movement now appears as it really is, moving obliquely upwards. If the triangle edges are explicit but irregular (Movie 7(b)), induced edge movement also vanishes, but texture expansion is less affected. This is an important control because it highlights the distinction between the expansion of the texture per se and the movement induced in the edges.

**Movie 7.** Illusory lateral movement of the oblique edges is cancelled by a local mask perforated with circles (Movie 7(a)), whilst within the circles, texture movement appears as it really is, moving obliquely upwards. Small irregularities in the edges also cancel the illusion (Movie 7(b)).

The optical flow seen in these movies would be extremely unusual in everyday visual experience. Might the brain be interpreting the anomalous optical flow as movement within a foreground object? Certainly, the illusory movement of the edges is associated with their being seen as figure rather than ground in Movie 8.

**Movie 8.** A paradox: lower down, the triangle appears as if expanding on a dark ground, but higher up the texture appears as if seen through a window, whose vertical edges appear static.

The induced expansion is not a general phenomenon but depends on the edges being obliquely aligned with the local motion. When the oblique edges in the top half of the array are made vertical, the texture they enclose appears as if seen through a window, and the expansion effect disappears. The whole pattern is thus paradoxical, because the oblique edges in the bottom half, and the texture within them, still appear as figure rather than ground, and do show expansion.

The illusions suggest unsuspected interactions at the edges of motion vector fields. Observers have also reported variation with fixation in these illusions, which might illuminate the role of eye movements in the integration of movement across complex textures.
